# Large Vaginal Varicosities in the Setting of Pregnancy without Known Hepatic or Vascular Risks: A Case Report and Review of the Literature

**DOI:** 10.1155/2018/2394695

**Published:** 2018-01-22

**Authors:** Mark Sueyoshi, Steven Clevenger, Elaine Hart

**Affiliations:** ^1^School of Medicine, University of California, Riverside, CA, USA; ^2^College of Osteopathic Medicine, Western University of Health Sciences, Pomona, CA, USA; ^3^School of Medicine, Loma Linda University, Loma Linda, CA, USA

## Abstract

Pregnancy may cause the onset of vaginal or vulvar varicosities that may be a concern for hemorrhage risk during childbirth. A 38-year-old female G4P1112 at 34 weeks and 1 day was referred to an outpatient OB/Gyn clinic for evaluation of a large vaginal mass. The referring provider had concern for malignancy. Lesions of the vulva were biopsied and found to be benign. For two months prior to presentation, she was experiencing discomfort with walking, yellow vaginal discharge, and dysuria. Treatment with fluconazole showed no improvement. She denied any personal or family history of malignancies, varicosities, or hepatic issues. Past surgical history was significant for laparoscopic cholecystectomy and two cesarean sections. A large vaginal mass during pregnancy is a concern whether it is malignancy or large vaginal varicosities that may put the patient at risk of severe hemorrhage during childbirth. We concluded that the mass was large vaginal varicosities as there was no discernible etiology. A repeat cesarean section was recommended due to the risk of hemorrhage during childbirth. For long-term management, close observation postpartum was recommended. Spontaneous resolution is a potential outcome and this is what our patient experienced. Without an underlying etiology, supportive measures are the best options.

## 1. Introduction

Vaginal varicosities are part of a larger set of complications that can occur as a result of venous congestion and obstruction in pregnant and nonpregnant patients alike. This can lead to varicosities anywhere in the genital and pelvic region. The occurrence of genital varicosities in pregnancy is uncommon with vulvar varicosities occurring in 2–4% of pregnancies and vaginal varicosities being even less common [1, 2]. One sample of 4676 patients seen in the St Mary's Hospital vein clinic showed that 4% of pregnant patients had perivulvar varicosities [3]. They usually develop after 12–26 weeks of pregnancy and largely self-resolve shortly after delivery [4–6]. Nonpregnancy-related causes of genital varicosities include portal hypertension and Klippel-Trenaunay syndrome [7–10]. Vaginal varicosities can also be part of a larger syndrome called pelvic congestion syndrome which often presents in pregnant patients as a constellation of pelvic pain, dyspareunia, dysmenorrhea, dysuria, vulvar, and perivulvar varicosities [1, 11, 12]. Although generally small, vaginal and genital varicosities can become large enough that concern over rupture and subsequent hemorrhage during vaginal birth has been raised by some practitioners [4, 13]. This has led some physicians to utilize cesarean section in selected cases to avoid the risk of hemorrhage, although due to limited reports the utility of this approach is not known [13, 14]. In this case we present large vaginal varicosities in a 38-year-old G4P1112 at 34 weeks and 1 day of gestation varicosities for which hemorrhage was a concern.

## 2. Case Presentation

A 38-year-old female G4P1112 at 34-week and 1-day gestational age dated by last menstrual period presented to the outpatient clinic for her first obstetrical visit. She was referred for evaluation of a large vaginal mass by an outside provider over concern of potential malignancy. There were also vulvar lesions which were punch biopsied showing squamous vaginal mucosa with some reactive cellular changes. A transvaginal ultrasound detailed the presence of varicosities within the vaginal wall. The patient had no prior history of vaginal varicosities. Her obstetrics history was significant for 2 prior C-sections, 1 ectopic pregnancy, and diet-controlled gestational diabetes. The patient had been complaining for the past 2 months of yellow vaginal discharge, dysuria, and discomfort made worse with walking. She had been treated unsuccessfully with fluconazole for this pain in the past. Fetal anatomy survey at 26 weeks and 2 days demonstrated no maternal anomalies of the cervix. The fetus was found to have mild bilateral hydronephrosis at 4 mm.

Our patient began menarche at age 12 and has been regular with a moderate flow lasting approximately 3 days. She has no known history of gynecologic diseases; STI screening with her last pap smear and HPV test were negative for abnormalities in October 2016. She has no history of past varicosities, coagulopathies, or hepatic disease. The patient had a significant past surgical history of laparoscopic cholecystectomy and hernia repair in 2014. She denied any personal or family history of cancer.

**Figure 1:**
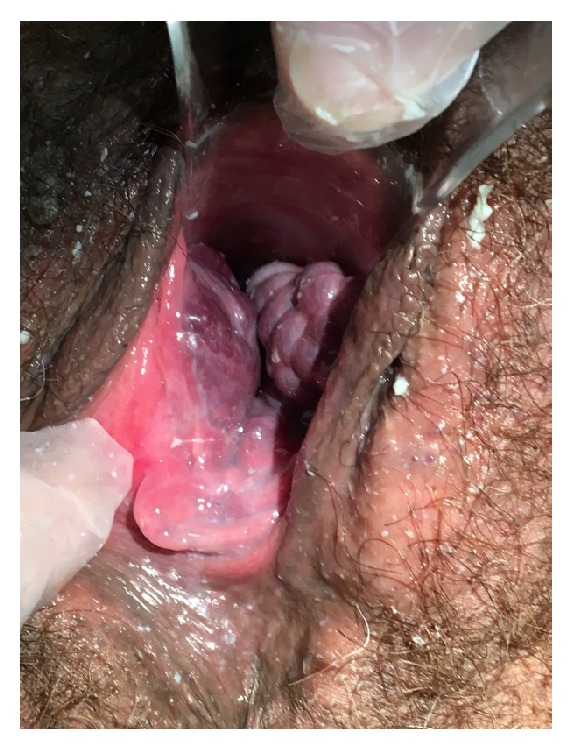
Vaginal varicosities protruding beyond hymenal ring.

**Figure 2:**
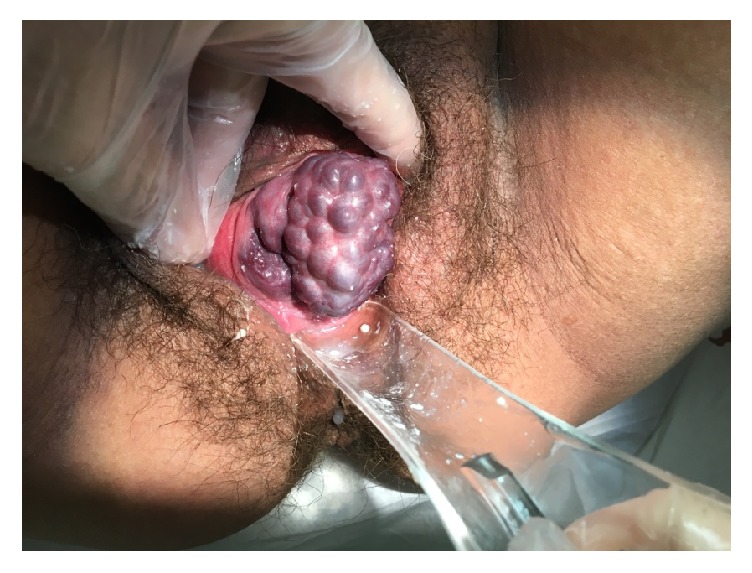
Vaginal Varicosities exposed during vaginal speculum exam.

On physical exam, significant anterior and lateral vaginal wall clusters of soft varicosities were noted. The varicosities filled the vagina and protruded beyond the hymenal ring (Figures 1 and 2). A second opinion obtained by a Gyn-Onc physician confirmed large vaginal varicosities by clinical exam. The patient was sent back to referring provider to continue prenatal care. The oupatient OB/Gyn consulting physician recommended repeating C-section due to both a history of 2 prior C-sections and the potential for hemorrhage if the patient were to attempt vaginal delivery. We did not perform further diagnostic testing for varicosities of the esophagus or a Doppler ultrasound of the extremities. As is typical with vulvar varicosities, our patient had complete resolution of her vaginal varicosities by her 6-week postpartum exam.

## 3. Discussion and Conclusions

While vulvar varicosities are fairly common in pregnancy, vaginal varicosities are much less common. Much of the literature focuses on vulvar varicosities during pregnancy, usually with spontaneous resolution within six weeks of delivery [15]. Treatment of these varicosities is generally conservative and symptomatic using a pelvic supporter for vulvar compression, support hose, leg elevation, minimizing sitting and standing, and exercise [16]. Several of the reported cases of vaginal varicosities are associated with underlying venous congestion due to portal hypertension [2, 10, 17]. However, vaginal varicosities have rarely been reported, with only 10 reported cases between 1967 and 2016 which could be found in general reviews of the literature [1, 2, 6, 10, 13, 17, 18]. Vaginal varicosities are believed to be rare due to the number of outlets for venous flow via venous plexuses [10]. The uterus and vagina both have their respective venous plexuses that drain into the hypogastric veins [10]. Orlando et al. hypothesize that the loss of uterine venous plexus, such as in the case of a hysterectomy, creates a situation in which congestion of the vaginal network could occur creating an ideal environment for varicosities formation [2].

Pregnancy itself causes several physiologic changes that favor varicosities formation. A study by Gant et al. demonstrated that pregnancy normally leads to acquired vascular refractoriness to Angiotensin II (ATII) [19]. Prostacyclin (PGI2) which has been implicated in angiotensin resistance during normal pregnancy is increased during late pregnancy [20]. The femoral venous pressure rises gradually from approximately 8 mmHg at the beginning of pregnancy to approximately 24 mmHg at term [21]. However, antecubital venous pressure does not change [21]. These asymmetrical venous pressure changes are likely a product of IVC compression by the growing fetus. The venous blood of the pelvis drains mainly through three pathways: internal iliac vein, femoral vein, and ovarian vein. Incompetence of the femoral vein is the most common cause for vulvar varicosities in nonpregnant women [12]. However, in pregnant women decreased pelvic venous return and IVC occlusion due to the enlarging uterus contribute to the formation of vulvar and vaginal varicosities [22]. The combination of physiologic ATII resistance of the vasculature and increased pressure caused by a growing uterus could allow for significant venous congestion and varicosities formation.

Vaginal varicosities can also occur due to diseases of hepatic origin or from a rare condition known as Klippel-Trenaunay syndrome, both of which can be further exacerbated during pregnancy. Klippel-Trenaunay syndrome is a rare disease, occurring in approximately 1 in 30000 live births. It is characterized by a triad of capillary malformations, vascular anomalies, and hypertrophy of bony and soft tissues. It can also present as varicosities in variable locations [23, 24]. Hepatic diseases that induce portal hypertension such as liver cirrhosis, NASH, and chronic hepatitis can contribute to varicosities formation [10]. However our patient did not exhibit any prior symptoms of this Klippel-Trenaunay syndrome nor did she have a history of hepatic disease, making these two conditions unlikely explanations for her varicosities.

Since vulvar varicosities are likely to resolve 6 weeks postpartum, it may be reasonable to assume that vaginal varicosities are also likely to resolve spontaneously; thus conservative management was taken before other interventions were made. Treatment options for vulvar varicosities include sclerotherapy or local excision for mild vulvar varicosities [5, 11, 12]. However, there is little information on the management of vaginal varicosities that do not self-resolve or are not due to a hepatic cause. Orlando et al. reviewed 6 cases of vaginal varicosities and their subsequent treatment. One of the cases had liver disease with resultant portal hypertension and three had known histories of total radical hysterectomy suggesting lack of uterine vascular network leading to increased venous pressure in the vaginal venous plexus. Four of the patients made complete recoveries by 34 months following a variety of treatments including partial vaginectomy, transvaginal ligation, and tamponade followed by balloon-occluded retrograde obliteration, tamponade, and surgical hemostasis followed by TIPS, and TIPS was followed by liver transplantation [2]. As for the other two cases, one died due to alcohol intoxication and the other from perioperative death [2].

The most likely and significant potential complications from our patient's vaginal varicosities include hemorrhage and thromboses during delivery. Several case reports have discussed bleeding from vaginal varicosities with etiologies rooted in portal hypertension [10, 18]. Kikuchi et al. documented a case of massive vaginal bleeding in a patient with vaginal varicosities after delivery of a 2562 g male infant with resultant hypotension of 64/35 mmHg treated with vaginal packing and blood transfusion [13]. Similarly, McHugh et al. reported a case of massive hemorrhage from vaginal varicosities in a nonpregnant 58-year-old patient suffering nonalcoholic steatohepatitis successfully treated with aggressive resuscitation and liver transplant [10]. In contrast to this, Furuta et al. noted that in spite of the concern over potential hemorrhage, the risk appears to be minor due to the shrinking and compression of vulvovaginal varicosities during the second stage of labor by the descending fetal head [4]. In spite of the potentially minimal risk of hemorrhage during vaginal delivery, it was decided that our patient's baby will be delivered via C-section. Her prior history of multiple C-sections and the presence of massive vaginal varicosities made the option for vaginal birth too risky. Vaginal varicosities can pose a risk to pregnant patients wanting to deliver vaginally due to the risk of hemorrhage, although by themselves vaginal varicosities are not significantly hazardous. Providers should consider this small risk in conjunction with other risk factors in their patients when deciding whether to attempt vaginal delivery.

## Abbreviations

C-section: Cesarean section
TIPS: Transjugular intrahepatic portosystemic shunt
ATII: Angiotensin II
PGI2: Prostaglandin I2
IVC: Inferior vena cava
STI: Sexually transmitted infection
HPV: Human papilloma virus
NASH: Nonalcoholic fatty liver disease.
